# Predicting Off-Target Binding Profiles With Confidence Using Conformal Prediction

**DOI:** 10.3389/fphar.2018.01256

**Published:** 2018-11-06

**Authors:** Samuel Lampa, Jonathan Alvarsson, Staffan Arvidsson Mc Shane, Arvid Berg, Ernst Ahlberg, Ola Spjuth

**Affiliations:** ^1^Pharmaceutical Bioinformatics Group, Department of Pharmaceutical Biosciences, Uppsala University, Uppsala, Sweden; ^2^Predictive Compound ADME and Safety, Drug Safety and Metabolism, AstraZeneca IMED Biotech Unit, Mölndal, Sweden

**Keywords:** target profiles, predictive modeling, conformal prediction, machine learning, off-target, adverse effects, workflow

## Abstract

Ligand-based models can be used in drug discovery to obtain an early indication of potential off-target interactions that could be linked to adverse effects. Another application is to combine such models into a panel, allowing to compare and search for compounds with similar profiles. Most contemporary methods and implementations however lack valid measures of confidence in their predictions, and only provide point predictions. We here describe a methodology that uses Conformal Prediction for predicting off-target interactions, with models trained on data from 31 targets in the ExCAPE-DB dataset selected for their utility in broad early hazard assessment. Chemicals were represented by the signature molecular descriptor and support vector machines were used as the underlying machine learning method. By using conformal prediction, the results from predictions come in the form of confidence *p*-values for each class. The full pre-processing and model training process is openly available as scientific workflows on GitHub, rendering it fully reproducible. We illustrate the usefulness of the developed methodology on a set of compounds extracted from DrugBank. The resulting models are published online and are available via a graphical web interface and an OpenAPI interface for programmatic access.

## 1. Introduction

Drug-target interactions are central to the drug discovery process (Yildirim et al., 2007), and is the subject of study for the field of chemogenomics (Bredel and Jacoby, 2004), which has emerged and grown over the last few decades. Drugs commonly interact with multiple targets (Hopkins, 2008), and off-target pharmacology as well as polypharmacology have important implications for drug efficacy and safety (Peters, 2013; Ravikumar and Aittokallio, 2018). Organizations involved in drug discovery, such as pharmaceutical companies and academic institutions, use many types of experimental techniques and assays to determine target interactions, including *in vitro* pharmacological profiling (Bowes et al., 2012). However, an attractive complementary method is to use computational (*in silico*) profiling of binding profiles for ligands (Cereto-Massagué et al., 2015), which also opens the possibility to predict hypothetical compounds. A common approach to the target prediction problem is to use a panel of structure-activity relationship (QSAR) models, with one model per target (Hansch, 1969), where chemicals in a knowledge base with known interaction values (numerical or categorical) are described numerically by descriptors, and a statistical learning model is trained to predict numerical values (regression) or categorical values (classification) for new compounds. The recent increase in the number of available SAR data points in interaction databases such as ChEMBL (Gaulton et al., 2017) and PubChem (Wang et al., 2017) makes it feasible to use ligand-based models to predict not only targets but also panels of targets. Several methods and tools are available for target prediction and for constructing and using target profiles. Bender et al. use a Bayesian approach to train models for 70 selected targets and use these for target profiling to classify adverse drug reactions (Bender et al., 2007). Chembench is a web-based portal, which, founded in 2008 is one of the first publicly available integrated cheminformatics web portals. It integrates a number of commercial as well as open source tools for dataset creation, validation, modeling and validation. It also supports building ensembles of models, for multiple targets (Walker et al., 2010; Capuzzi et al., 2017). The Online chemical modeling environment (OCHEM), is a web-based platform that intends to serve as multi-tool platform where users can select among the many available alternatives in terms of tools and methods, for all of the steps of creating a predictive model, such as data search, selection of descriptors and machine learning model, as well as assessment of the resulting model. OCHEM also encourages tool authors to contribute with their own tools to be integrated in the platform (Sushko et al., 2011). Yu et al. use Random Forest (RF) and Support Vector Machines (SVM) to predict drug-target interactions from heterogeneous biological data (Yu et al., 2012). TargetHunter (Wang et al., 2013) is another online tool that uses chemical similarity to predict targets for ligands, and show how training models on ChEMBL data can enable useful predictions on examples taken from PubChem bioassays. Yao et al. describe TargetNet (Yao et al., 2016), a web service for multi-target QSAR models; an online service that uses Naïve Bayes. The polypharmacology browser (Awale and Reymond, 2017) is a web-based target prediction tool that queries ChEMBL bioactivity data using multiple fingerprints.

We observe three important shortcomings among previous works. Primarily, available methods for ligand-based target profiling often do not offer valid measures of confidence in predictions, leaving the user uncertain about the usefulness of predictions. Secondly, the majority of the web tools lack an open and standardized API, meaning that it is not straightforward (and in most cases not possible at all) to consume the services programmatically, e.g., from a script or a scientific workflow tool such as KNIME (Mazanetz et al., 2012). Thirdly, previous works do not publish the pre-processing and modeling workflows in reproducible formats, rendering it hard to update the models as data changes, and limits the portability of methods. In fact, most implementations are only accessible from a website without the underlying implementations being openly available for inspection, which limits both the reproducibility (Stodden et al., 2016), and verifiability (Hinsen, 2018) of their implementation.

We here present an approach for ligand-based target profiling using a confidence framework, delivering target profiles with confidence scores for the predictions of whether a query compound interacts with each target. The confidence scores were calculated using the Conformal Prediction methodology (CP) (Vovk et al., 2005), which has been successfully demonstrated in several recent studies (Norinder et al., 2014, 2016; Cortés-Ciriano et al., 2015; Forreryd et al., 2018). For readers new to the CP methodology, we recommend (Gammerman and Vovk, 2007) for a good and gentle general overview, and Norinder et al. (2014) for a good introduction to CP for cheminformatics. The goal of this study was to create an automated and reproducible approach for generating a predicted target profile based on QSAR binding models, with the models making up the profile published online as microservices and the profile accessible from a web page. Although the models give a confidence measure we also set out to evaluate them on a test set to see how well they performed on representative data. We exemplified the process by creating a profile for the targets for broad early hazard assessment as suggested by Bowes et al. (2012).

## 2. Methods

### 2.1. Training data

We based this study upon data from the ExCAPE-DB dataset (Sun et al., 2017b). The reason for this is that ExCAPE-DB combines data about ligand-target binding from ChEMBL with similar data from PubChem, where importantly, PubChem contains many true non-actives, which has been shown earlier to result in better models than by using random compounds as non-actives (Mervin et al., 2015). The data in ExCAPE-DB has also gone through extensive filtering and pre-processing, specifically to make it more useful as a starting point for QSAR studies. For more details on the data filtering and processing done in the ExCAPE-DB dataset, we refer to Sun et al. (2017b).

A scientific workflow was constructed to automate the full data pre-processing pipeline. The first step comprises extracting data on binding association between ligands and targets from the ExCAPE-DB dataset (Sun et al., 2017b), more specifically the columns Gene symbol, Original entry ID (PubChem CID or CHEMBL ID), SMILES and Activity flag. This was performed early in the workflow to make subsequent data transformation steps less time-consuming, given the relatively large size of the uncompressed ExCAPE-DB data file (18 GB). From the extracted dataset, all rows for which there existed rows with a conflicting activity value for the same target (gene symbol) and SMILES string, were completely removed. Also, all duplicates in terms of the extracted information (Original entry ID, SMILES, and Activity flag) were replaced by a single entry, and thus deduplicated. Note that deduplication on InChI level was already done in for the ExCAPE-DB dataset in Sun et al. (2017b), but since the signatures descriptor is based on SMILES, which is a less specific chemical format than InChI (certain compounds that are unique in InChI might not be unique in SMILES) this turns out to have resulted in some duplicate and conflicting rows in terms of SMILES still appearing in the dataset. Since this is a potential problem in particular if the exact same SMILES end up in both the training and calibration or test set, we performed this additional deduplication, on the SMILES level^1^. For full information about the pre-processing done by the ExCAPE-DB authors, see Sun et al. (2017b). As a help to the reader we note that the activity flag is – in the ExCAPE-DB dataset—set to active (or “A”) if the dose-response value in the binding assays was lower than 10 μ*M* and non-active (or “N”) otherwise.

A subset of the panel of 44 binding targets as suggested in Bowes et al. (2012) was selected for inclusion in the study. The selection was based on the criteria that targets should have at least 100 active and at least 100 non-active compounds. In addition some targets were excluded for which data was not found in ExCAPE-DB. This is described in detail below. Some of the gene symbols used in Bowes et al. (2012) were not found in their exact form in the ExCAPE-DB dataset. To resolve this, PubMed was consulted to find synonymous gene symbols with the following replacements being done: *KCNE1* was replaced with *MINK1* which is present in ExCAPE-DB. *CHRNA1* (coding for the α1 sub-unit of the Acetylcholine receptor) was excluded, as it is not present in the dataset (*CHRNA4*, coding for the α4 sub-unit of the Acetylcholine receptor, is present in the dataset). We note though, that both *MINK1* and *CHRNA4* were removed in the filtering step mentioned above, since the dataset did not contain more than 100 active and 100 non-active compounds for *MINK1* nor *CHRNA*. However, since one aim of the study is to present and publish an automated and reproducible data processing workflow, these targets could potentially be included in subsequent runs on later versions of the database with additional data available.

The resulting dataset (named Dataset1) consists of 31 targets (marked as “included” in Table 1). For 21 of these targets, the dataset contained less than 10,000 non-active compounds, which makes them stand out from the other datasets, and where some of them contain a problematically low amount of non-actives. These 21 targets are referred to as Dataset2, and their respective target datasets were expanded with randomly selected examples from the ExCAPE-DB dataset which were not reported to be active for the target, thus being “assumed non-active.” These target datasets are marked with a ✓ in the “Assumed non-actives added” column of Table 1. The number of new examples was chosen such that the total number of non-actives and assumed non-actives added up to twice the number of actives, for each target, respectively. The compounds for the remaining 10 targets, which were not extended with assumed non-actives, were named Dataset3.

**Table 1:** The panel of targets used in this study, identified by gene symbol.

			**Non-actives**	**Non-actives**		
			**(before adding**	**(after adding**		
			**assumed non-actives**	**assumed non-actives**	**Assumed non-**	
	**Gene symbol**	**Actives**	**and deduplication)**	**and deduplication)**	**actives added**	**Remarks**
INCLUDED	ACHE	3,160	1,152	5,824	✓	
	ADORA2A	5,275	593	10,092	✓	
	ADRB1	1,306	149	2,544	✓	
	ADRB2	1,955	342,282	341,925		
	AR	2,593	4,725	4,866	✓	
	AVPR1A	1,055	321,406	321,098		
	CCKAR	1,249	132	2,458	✓	
	CHRM1	2,776	417,549	358,330		
	CHRM2	1,817	152	3,440	✓	
	CHRM3	1,676	144	3,234	✓	
	CNR1	5,336	400	10,220	✓	
	CNR2	4,583	402	8,676	✓	
	DRD1	1,732	356,201	355,909		
	DRD2	8,323	343,206	342,958		
	EDNRA	2,129	124	4,050	✓	
	HTR1A	6,555	64,578	64,468		
	HTR2A	4,160	359,962	359,663		
	KCNH2	5,330	350,773	350,452		
	LCK	2,662	283	5,246	✓	
	MAOA	1,260	1,083	2,452	✓	
	NR3C1	2,525	4,382	4,804	✓	
	OPRD1	5,350	826	9,580	✓	
	OPRK1	3,672	303,335	303,111		
	OPRM1	5,837	2,872	11,252	✓	
	PDE3A	197	110	392	✓	
	PTGS1	849	729	1,634	✓	
	PTGS2	2,862	827	5,162	✓	
	SCN5A	316	119	624	✓	
	SLC6A2	3,879	218	7,498	✓	
	SLC6A3	5,017	106,819	106,594		
	SLC6A4	7,228	382	13,660	✓	
NOT INCLUDED	ADRA1A	1,782	24			
	ADRA2A	839	39			
	CACNA1C	166	20			
	CHRNA1	–	–			Not in ExCAPE-DB
	CHRNA4	256	17			
	GABRA1	112	5			
	GRIN1	555	92			
	HRH1	1,218	65			
	HRH2	394	56			
	HTR1B	1,262	86			
	HTR2B	1,159	66			
	HTR3A	584	65			
	KCNQ1	37	303,466			
	MINK1	929	8			Synonym to KCNE1
	PDE4D	484	98			

*Actives and non-actives refer to the number of ligand interactions marked as active and non-active in ExCAPE-DB. The labels “included” and “not included” to the left, for the two row ranges, indicate whether targets did pass the filtering criteria of at least 100 actives and 100 non-actives, to be included*.

In order to validate the predictive ability of the trained models, a new dataset was created (Dataset4) by withholding 1,000 compounds from the ExCAPE-DB dataset, to form an external validation dataset. The compounds chosen to be withheld were the following: (i) all small molecules in DrugBank (version 5.0.11) with status “withdrawn,” for which we could find either a PubChem ID or a CHEMBL ID, (ii) a randomly selected subset of the remaining compounds in DrugBank 5.0.11, with status “approved,” for which we could also find PubChem or CHEMBL IDs, until a total number of 1,000 compounds was reached. No regard was paid to other drug statuses in DrugBank such as “investigational.”

**Figure 1:**
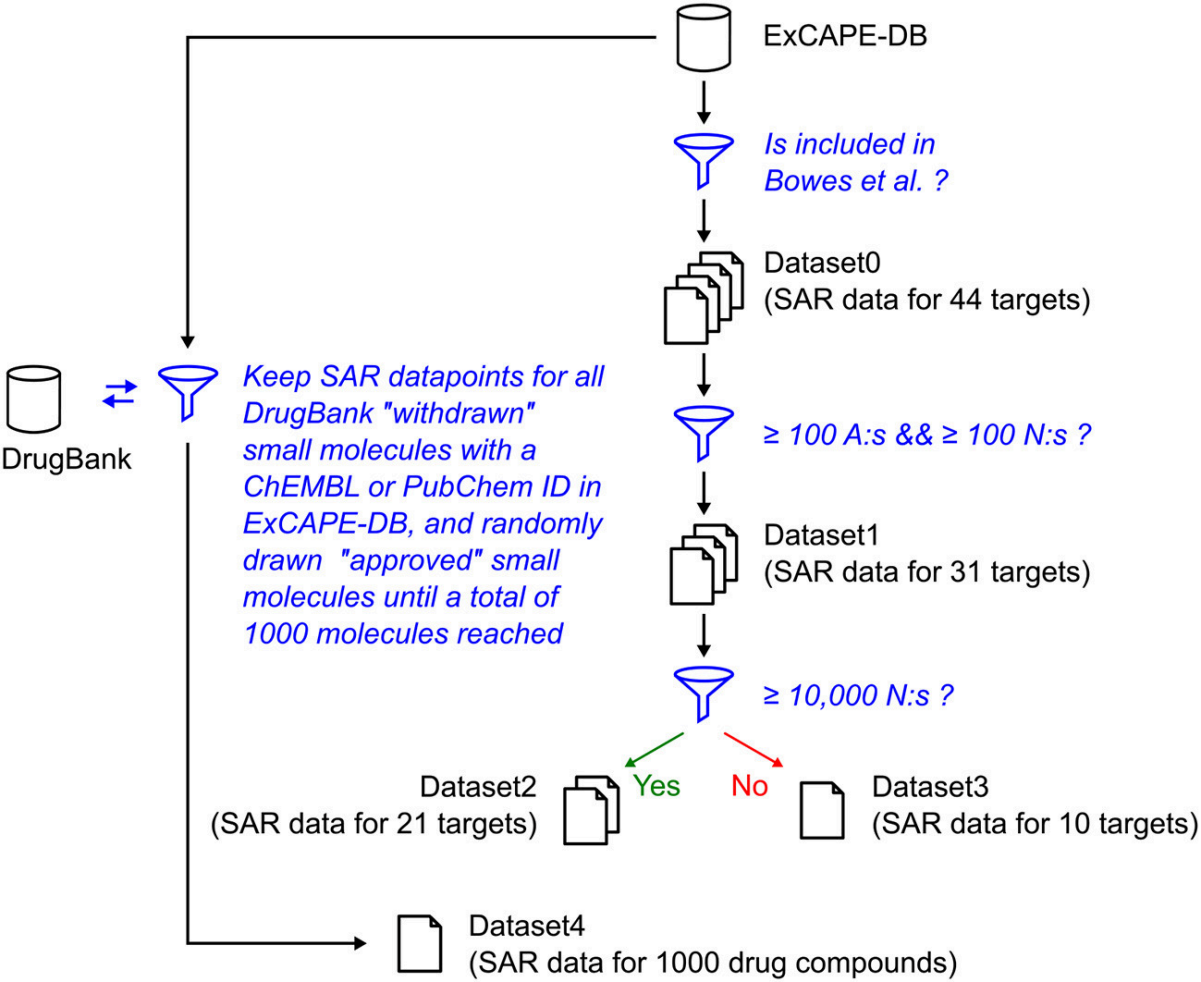
Graphical overview over how the raw datasets used in this study were created. The blue funnel symbol and text represent filtering steps, while the barrel and document symbols represent datasets. The criteria for the filtering steps are shown with blue text. “A” represents “Actives,” and “N” represents “Non-actives.”

The relation of the mentioned datasets Dataset1-4 are shown in a graphical overview of how they were created in Figure 1, and in Table 2, which summarizes in words how each dataset was created.

**Table 2:** Summary of datasets discussed.

**Name**	**Description**
Dataset0	SAR data points for all 44 targets in Bowes et al. (2012) which are available in ExCAPE-DB.
Dataset1	SAR data points for the 31 targets in Dataset0 for which there were at least 100 actives and 100 non-actives.
Dataset2	SAR data points for targets with least 10,000 non-actives.
Dataset3	SAR data points for targets which had less than 10,000 non-actives, thus the same as Dataset1 with Dataset2 excluded.
Dataset4	SAR points making up the external test, by extracting rows from ExCAPE-DB for a selected set of 1,000 compounds in DrugBank (All withdrawn, and randomly sampled approved, drugs, until reaching 1,000 drugs).

*See also Figure 1 for a graphical overview of how each dataset was created*.

The Conformal Prediction methodology, in particular with the Mondrian approach, can handle differing sizes of the datasets well (Norinder and Boyer, 2017), and so we see no reason to stick to the exact same number of compounds as the actives. Instead we use an active:non-active ratio of 1:2 between the classes. The justification for this is that the assumed non-actives likely have chemistry coming from a larger chemical space compared to the known compounds, thus by adding more of the assumed non-actives we can hopefully increase the number of examples in the regions of chemical space that are of interest for separating the two classes.

All the targets, with details about their respective number of active and non-active compounds, and whether they are included or not, are summarized in Table 1.

### 2.2. Conformal prediction

Conformal Prediction (CP) (Vovk et al., 2005) provides a layer on top of existing machine learning methods and produces valid prediction regions for test objects. This contrasts to standard machine learning that delivers point estimates. In CP a prediction region contains the true value with probability equal to 1 − ϵ, where ϵ is the selected significance level. Such a prediction region can be obtained under the assumption that the observed data is exchangeable. An important consequence is that the size of this region directly relates to the *strangeness* of the test example, and is an alternative to the concept of a model's *applicability domain* (Norinder et al., 2014). For the classification case a prediction is given as set of conformal *p-values*^2^, one for each class, which represent a ranking for the test object. The *p*-values together with the user decided ϵ produces the final prediction set. Conformal Predictors are Mondrian, meaning that they handle the classes independently, which has previously been shown to work very well for imbalanced datasets and remove the need for under/oversampling, boosting or similar techniques (Norinder and Boyer, 2017; Sun et al., 2017a).

Conformal Prediction as originally invented, was described for the online transductive setting, meaning that the underlying learning model had to be retrained for every new test object. Later it was adapted for the off-line inductive setting too, where the underlying model is trained only once for a batch of training examples. The Inductive Conformal Predictor (ICP), which is used in this study, require far less computational resources, but has the disadvantage that a part of the training set must be set aside as a *calibration set*. The remaining data, called *proper training set*, is used to train the learning model. As the partitioning of data into a calibration set and proper training set can have a large influence on the performance of the predictor, it is common to redo this split multiple times and train an ICP for each such split. This results in a so called Aggregated Conformal Predictor (ACP) that aggregates the predictions for each individual ICP.

In this study we used the Mondrian ACP implementation in the software CPSign (Arvidsson, 2016), leveraging the LIBLINEAR SVM implementation (Fan et al., 2008) together with the signatures molecular descriptor (Faulon et al., 2003). This descriptor is based on the neighboring of atoms in a molecule and has been shown to work well for QSAR studies (Alvarsson et al., 2016; Lapins et al., 2018) and for ligand-based target prediction (Alvarsson et al., 2014). Signatures were generated with height 1-3, which means that molecular sub-graphs including all atoms of distance 1, 2, or 3 from initial atoms, are generated. Support vector machines is a machine learning algorithm which is commonly used in QSAR studies (Norinder, 2003; Zhou et al., 2011) together with molecular signatures and similar molecular descriptors, e.g., the extended connectivity fingerprints (Rogers and Hahn, 2010). As nonconformity measure we used the distance between the classifier's decision surface and the test object, as previously described by Eklund et al. (2015). In order to not use the assumed non-active compounds in Dataset2 in the calibration set of the ICPs, these additional compounds were treated separately, by providing them to the CPSign software with the ––proper-train parameter, see the CPSign documentation (Arvidsson, 2016). By using this parameter the additional compounds are only added to the proper training set, thus being used for training the underlying SVM model, but not for the calibration of the predictions. This ensures that potentially non-typical chemistry in the additional assumed non-active compounds does not affect the calibration of the predictions in a negative way.

### 2.3. Hyper-parameter tuning

For each of the 31 targets in Dataset1, a parameter sweep was run to find the optimal value of the cost parameter of LIBLINEAR, optimizing modeling efficiency using 10-fold cross validation. The training approach used an Aggregated Conformal Predictor (ACP) with 10 aggregated models. The parameter sweep evaluated three values for the cost parameter for each target; 1, 10, and 100. The efficiency measure used for the evaluation was the observed fuzziness (OF) score described in Vovk et al. (2016) as:

(1) $$\begin{array}{l} OF=\frac{1}{m}\overset{m}{\underset{i=1}{\sum }}\underset{y_{i}\neq y}{\sum }p_{i}^{y}, \end{array}$$

where $p_{i}^{y}$ is the *p*-value of the *ith* test case for class *y*, and *m* is the number of test examples, or in our case with only two classes:

(2) $$\begin{array}{l} OF=\frac{\;\underset{\begin{array}{c} i,\;y_{i}=A \end{array}}{\sum }p_{i}^{N}+\underset{\begin{array}{c} i,\;y_{i}=N \end{array}}{\sum }p_{i}^{A}}{m_{A}+m_{N}} \end{array}$$

where $p_{i}^{N}$ is the *ith* *p*-value for class *N*, $p_{i}^{A}$ is the *ith* *p*-value for class *A* and *m*_*A*_ and *m*_*N*_ is the number of test examples in class *A* and *N*, respectively. *OF* is basically an average of the *p*-values for the wrong class, i.e., lower fuzziness means better prediction.

To study the effect of imbalanced datasets on efficiency, we also implemented a modified version of *OF*, due to the fact that *OF* is influenced more by values in the larger class in case of imbalanced datasets, referred to as *class-averaged observed fuzziness* (*CAOF*) as:

(3) $$\begin{array}{l} CAOF=\frac{\;\underset{\begin{array}{c} i,\;y_{i}=A \end{array}}{\sum }p_{i}^{N}}{m_{A}}+\frac{\;\underset{\begin{array}{c} i,\;y_{i}=N \end{array}}{\sum }p_{i}^{A}}{m_{N}} \end{array}$$

with the same variable conventions as above. Where *OF* is only an average for the *p*-values in the test set, *CAOF* averages the contribution from each class separately, meaning that for very imbalanced cases *OF* is mostly affected by the larger class, while for *CAOF*, both classes contribute equally much, regardless of their respective number of *p*-values. *CAOF* was not used for cost selection, but is provided for information in the results from the workflow.

A commonly used efficiency measure in CP is the size of the prediction region or set given by the predictor. In the classification setting, this is expressed as the fraction of *multi-label* predictions. This measure is denoted as the *M criterion* (MC) and described in Vovk et al. (2016):

(4) $$\begin{array}{l} \text{M criterion}=\frac{1}{m}\overset{m}{\underset{i=1}{\sum }}1_{\left\{|\Gamma_{i}|>1\right\}} \end{array}$$

where **1**_*E*_ denotes the indicator function of event *E*, returning the value 1 if *E* occurs and 0 otherwise, and Γ_*i*_ denotes the prediction set for test example *i*. A smaller value is preferable.

### 2.4. Modeling workflow

**Figure 2:**
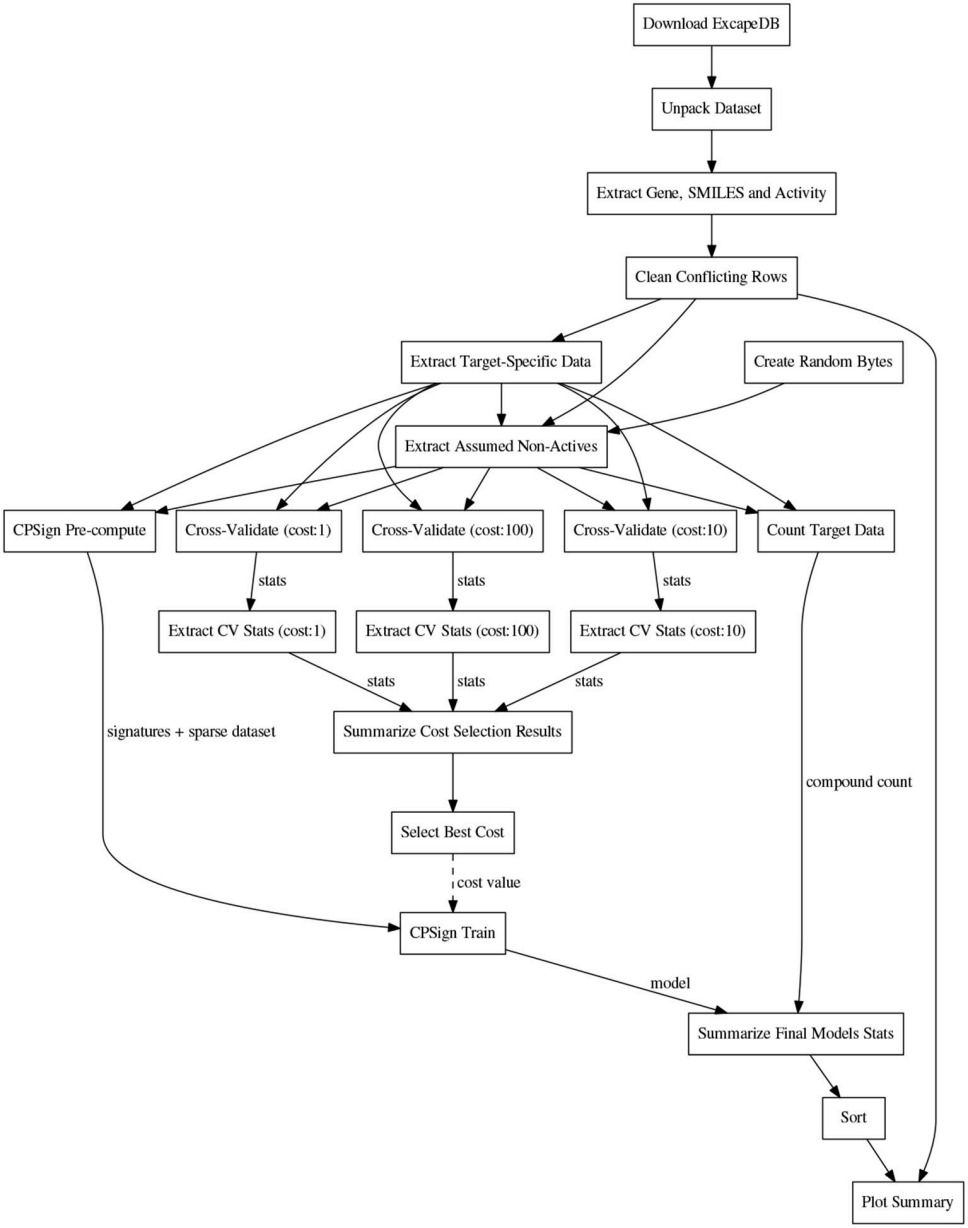
Schematic directed graph of processes and their data dependencies in the modeling workflow used in the experiments in this study. Boxes represent processes, while edges represent data dependencies between processes. The direction of the edges show in which direction data is being passed between processes. The order of execution is here from top to bottom, of the graph. Each experiment contains additions and modifications to the workflow, but the workflow shown here, exemplifies the basic structure, common among most of the workflows. For more detailed workflow plots, see Supplementary Data Sheet 1, Figures S4, S5.

Before the training, the CPSign precompute command was run, in order to generate a sparse representation of each target's dataset. ACPs consisting of 10 models were then trained for each target using the CPSign train command. The cost value used was the one obtained from the hyper-parameter tuning. The observations added as “assumed non-actives” were not included in the calibration set to avoid biasing the evaluation. The computational workflows for orchestrating the extraction of data, model building, and the collection of results for summarizing and plotting were implemented in the Go programming language using the SciPipe workflow library that is available as open source software at scipipe.org (Lampa et al., 2018b). The cost values for each target are stored in the workflow code, available on GitHub (PTP, 2018). A graphical overview of the modeling workflow is shown in Figure 2. More detailed workflow graphs are available in Supplementary Data Sheet 1, Figures S4, S5.

### 2.5. Model validation

The models built were validated by predicting the binding activity against each of the 31 targets for all compounds for which there existed known binding data for a particular target in ExCAPE-DB. The validation was done with CPSign's validate command, predicting values at confidence levels 0.8 and 0.9.

## 3. Results

### 3.1. Published models

Models for all targets in Dataset1 were produced in the form of portable Java Archive (JAR) files, which were also built into similarly portable Docker containers, for easy publication as microservices. The model JAR files, together with audit log files produced by SciPipe, containing execution traces of the workflow (all the shell commands and parameters) used to produce them, are available for download at Lampa et al. (2018a). The models can be run if obtaining a copy of the CPSign software and a license, from Genetta Soft AB.

### 3.2. Validity of models

**Figure 3:**
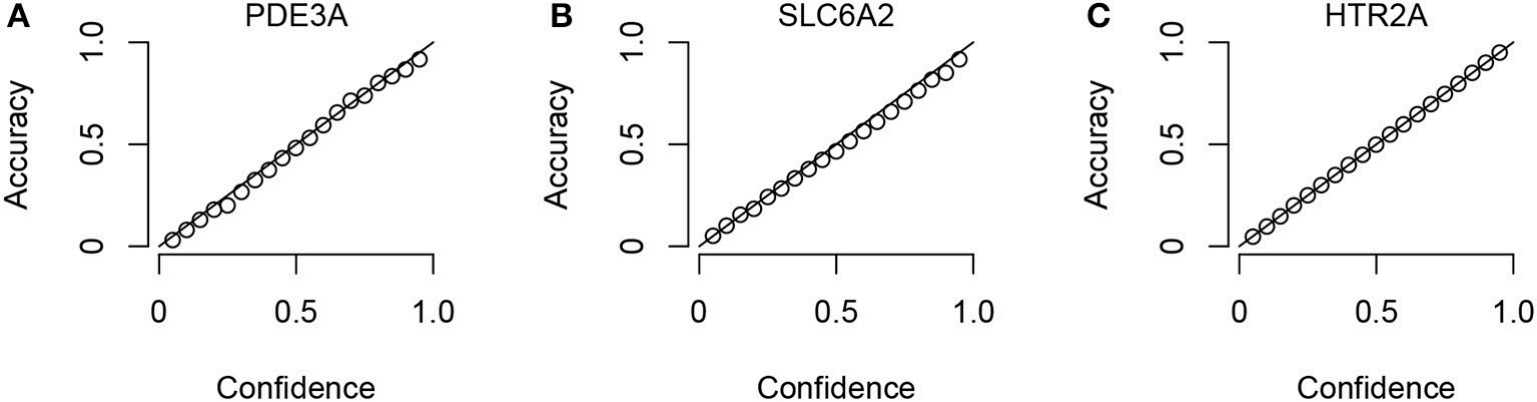
Three representative calibration plots, for models PDE3A **(A)**, SLC6A2 **(B)**, and HTR2A **(C)**, based on the smallest, the median, and the largest target data sets in terms of total number of compounds. The plots show accuracy vs. confidence, for the confidence values between 0.05 and 0.95 with a step size of 0.05.

To check that the Conformal Prediction models are valid (i.e., that they predict with an error rate in accordance to the selected significance level), calibration plots were generated in the cross validation step of the workflow. Three example plots, for three representative targets (the smallest, the median-sized and the largest, in terms of compounds in ExCAPE-DB) can be seen in Figure 3, while calibration plots for all targets can be found in the Supplementary Data Sheet 1 (Figure S1). From these calibration plots we conclude that all models produce valid results over all significance levels.

### 3.3. Efficiency of models

**Figure 4:**
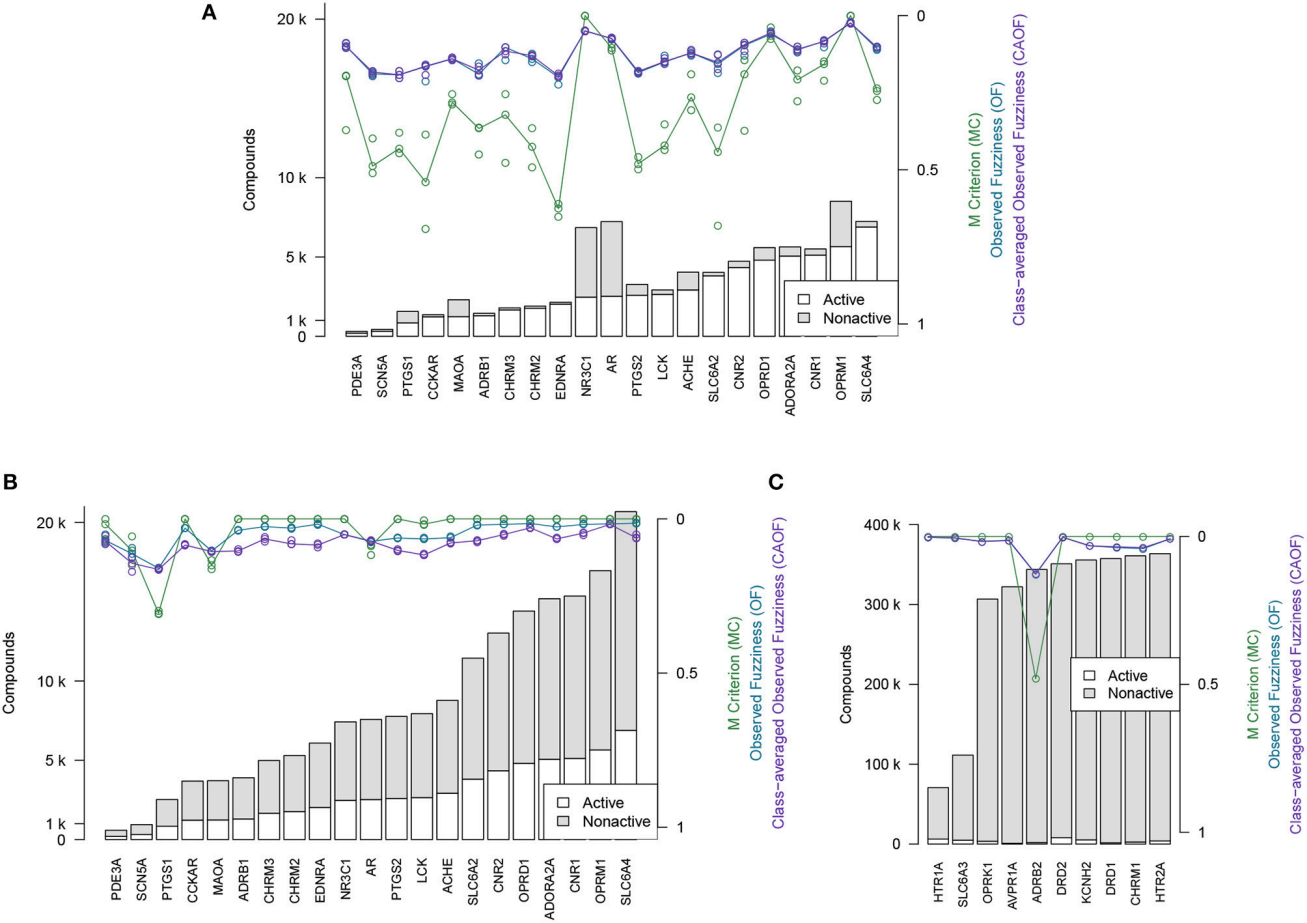
Efficiency metrics (M Criterion, Observed Fuzziness and Class-Averaged Observed Fuzziness) for Dataset1, Dataset2, and Dataset3. **(A)** Dataset2 without extending with assumed non-actives. Circles show individual results from the three replicate runs that were run, while the lines show the median value from the individual replicate results. Targets are here sorted by number of active compounds. **(B)** Dataset2 after extending with assumed non-actives. Circles show individual results from the three replicate runs that were run, while the lines show the median value from the individual replicate results. Targets are here sorted by number of active compounds. **(C)** Dataset3, the 10 largest target datasets, which were not extended with assumed non-actives. Targets are here sorted by total number of compounds.

The efficiency metrics OF, CAOF and MC for Dataset2 (without adding assumed non-actives) are shown in Figure 4A. In Figure 4B, the same metrics are shown for when all target datasets in Dataset2 have been extended with assumed non-actives, to compensate for these datasets' relative low number of non-actives. We observe that by adding assumed non-actives for datasets with few non-actives, we improve the efficiency of models trained on these datasets. Thus, this strategy of extending the “small” target datasets in Dataset2 was chosen for the subsequent analysis workflows.

### 3.4. External validation

**Figure 5:**
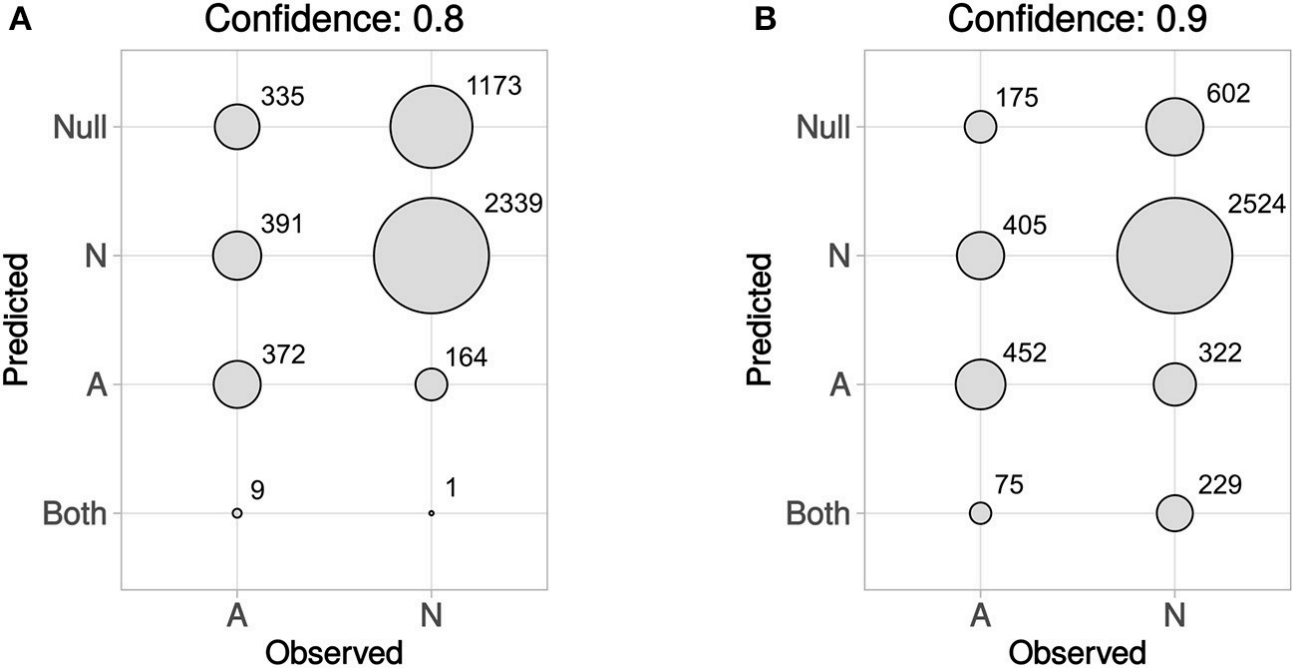
Predicted vs. observed labels, for all targets, for the prediction data, at confidence level 0.8 **(A)** and 0.9 **(B)**. “A” denotes active compounds, and “N” denotes non-active compounds. The x-axis show observed labels (as found in ExCAPE-DB), while the y-axis show the set of predicted labels. The areas of the circles are proportional to the number of SAR data points for each observed label/predicted label combination. For predicted vs. observed labels for each target individually, see Supplementary Data Sheet 1, Figures S2, S3.

In Figure 5 predicted vs. observed labels for Dataset4 is shown, for confidence levels 0.8 and 0.9, respectively. See the methods section and in particular Figures 1, 2, for information about how Dataset4 was created. “A” denotes active compounds and “N” denotes non-active ones. It can be seen how the number of prediction of “Both” labels increase when the confidence level increases from 0.8 to 0.9. This is as expected, as this means that fewer compounds could be predicted to only one label, with the higher confidence level. The number of “Null” predictions decreases at the higher confidence, which is also as expected. The reason is that with a higher confidence, the predictor must consider less probable (in the Conformal Prediction ranking sense) predictions to be part of the prediction region. This behavior might seem backwards, but at a higher confidence the predictor has to include less likely predictions in order to reach the specified confidence level, which leads to larger prediction sets. For predicted vs. observed labels for each target individually, see Supplementary Data Sheet 1, Figures S2, S3. Because of the fact that CP produces sets of predicted labels, including Null, and Both in this case, the common sensitivity and specificity measures do not have clear definitions in this context. Because of this, we have not included calculated values for them but have instead included compound counts for the predicted label sets in Figure 5 summarized for all targets, and as CSV files in Supplementary Data Sheet 2 (for 0.8 confidence) and 3 (for 0.9 confidence), for each target specifically.

### 3.5. Target profile-as-a-service

**Figure 6:**
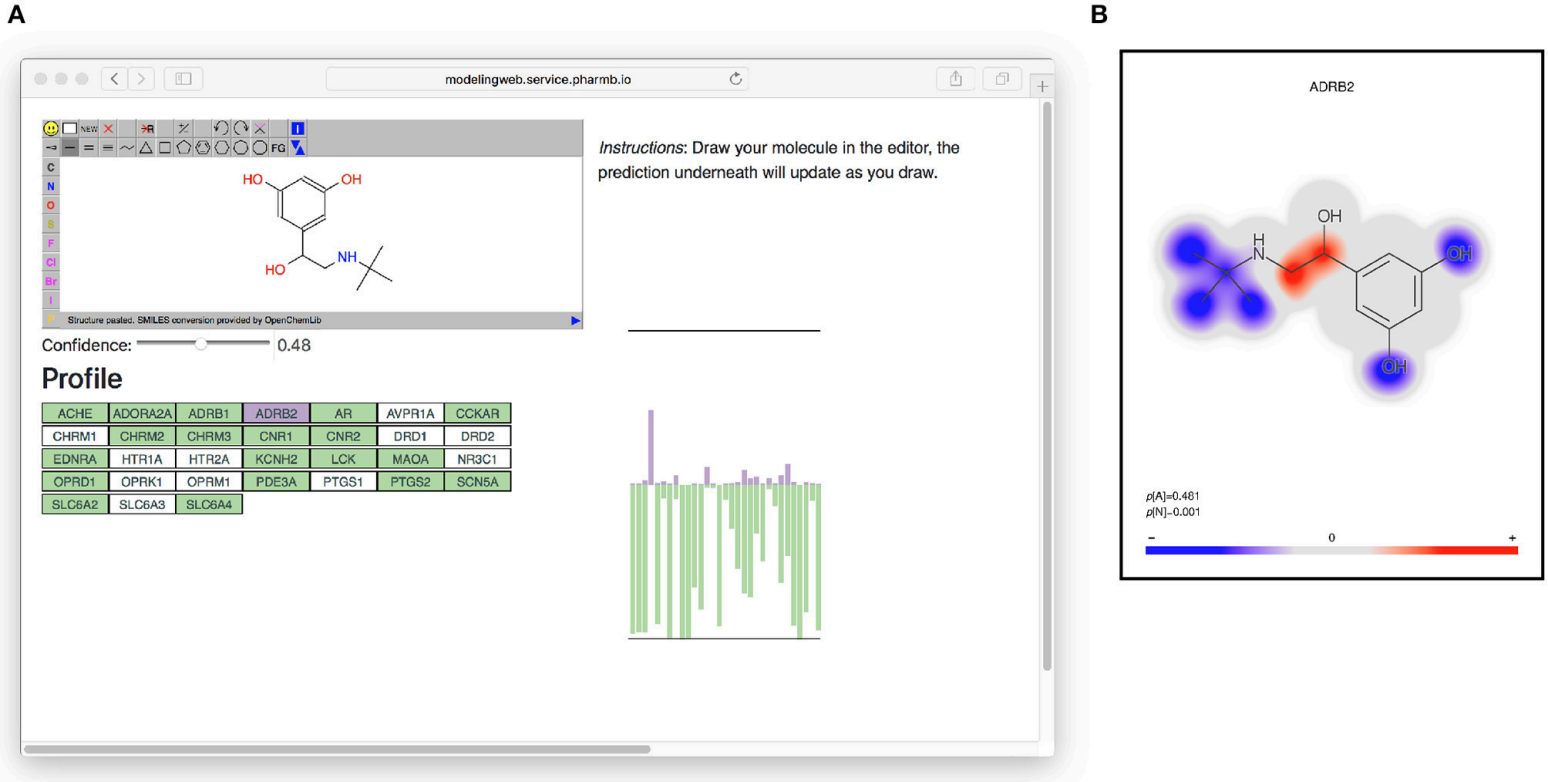
The prediction profile for Terbutaline, a known selective beta-2 adrenergic agonist used as a bronchodilator and tocolytic. **(A)** The profile as seen on the web page (on the right hand in the figure). To show the profile, the user draws a molecule and selects a confidence level, whereafter the profile will update underneath. The profile is shown as a bar plot with two bars for each target: A purple bar, pointing in the upward direction, indicating the size of the *p*-value of the “Active” label, and a green bar, pointing downwards, indicating the size of the *p*-value for the “Non-active” label. **(B)** Coloring of which parts of the molecule contributed the most to the prediction for ADBR2. Red color indicates the centers of molecular fragments (of height 1–3) that contributed most to the larger class, while blue color indicates center of fragments contributing most to the smaller class. In this case the larger class is “Active,” which can be seen in the size of the *p*-values in the bottom left of the figure (p[A] = 0.481 >p[N] = 0.001).

All models based on Dataset2 were published as microservices with REST APIs publicly made available using the OpenAPI specification (Ope, 2018a) on an OpenShift (Ope, 2018b) cluster. A web page aggregating all the models was also created. The OpenAPI specification is a standardization for how REST APIs are described, meaning that there is a common way for looking up how to use the REST API of a web service and that greatly simplifies the process of tying multiple different web services together. It simplifies calling the services from scripts as well as from other web pages, such as the web page (Figure 6) that generates a profile image out of the multiple QSAR models. At the top of the web page (see Figure 6) is an instance of the JSME editor (Bienfait and Ertl, 2013) in which the user can draw a molecule. As the user draws the molecule, the web page extracts the SMILES from the editor and sends it to the individual model services to get predictions based on all available models. The user can set a threshold for the confidence and get visual feedback on whether the models predict the drawn molecule as active or non-active for each of the targets, at the chosen confidence level. In Figure 6 on the right side is a graphical profile in the form of a bar plot where confidence of the active label is drawn in the upward direction and the confidence for non-active is drawn in the downward direction. Hovering over a bar in the plot will give information about which model the bar corresponds to. The web page can be accessed at http://ptp.service.pharmb.io/.

### 3.6. Example predictions

**Figure 7:**
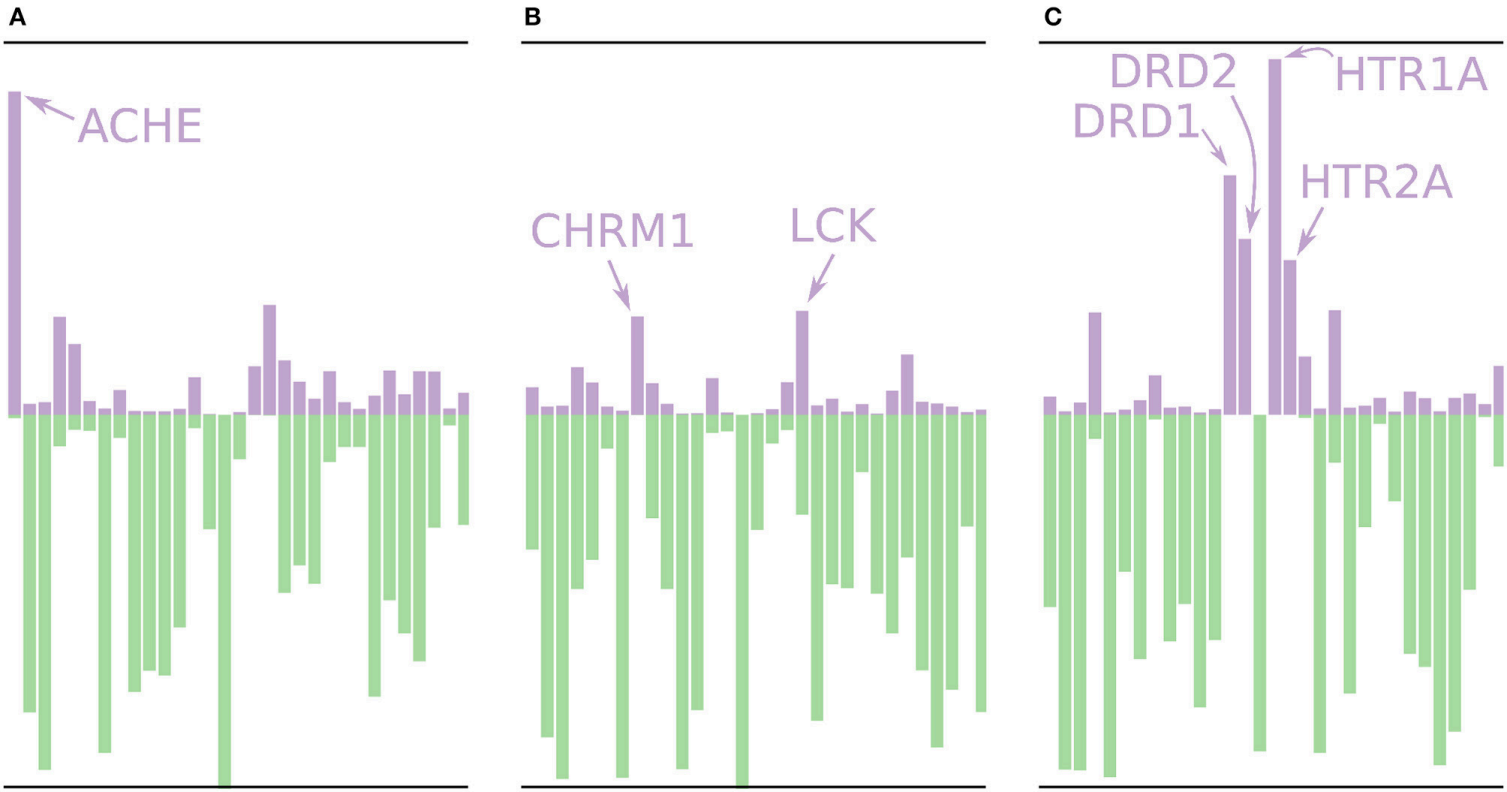
Profiles for a few of the removed drugs using the validation models, i.e., these molecules are not in the training sets for the models. The profiles are shown as bar plots with two bars for each target: A purple bar pointing in the upward direction, indicating the size of the *p*-value of the “Active” label, and a green bar pointing downwards, indicating the size of the *p*-value for the “Non-active” label. **(A)** The profile for Tacrine, a centrally acting anticholinesterase, with a distinct peak for the ACHE gene. **(B)** The profile for Pilocarpine, a muscarinic acetylcholine receptor M_1_ agonist, with only two moderately higher peaks for active prediction, CHRM1 and LCK. **(C)** The profile for Pergolide, a DRD1, DRD2, HTR1A, and HTR2A agonist, which is reflected by the four highest *p*-values for an active prediction.

Using the models built without the external validation dataset (Dataset4), target profiles were predicted for three molecules from the test set (Figure 7), i.e., the profiles were made for drugs that the models have not seen before. Figure 7A shows the target profile for Tacrine, a centrally acting anticholinesterase, with a distinct peak for the ACHE gene, as expected. Further, we note that most other targets are predicted as non-active with high *p*-values (green color) or predicted as active with relatively low *p*-values (purple color). Figure 7B shows the target profile for Pilocarpine, a muscarinic acetylcholine receptor M_1_ agonist, with a target profile consisting of mostly non-active predictions, and only two mildly active targets (CHRM1 and LCK). We note that LCK has a similar *p*-value for active and non-active. For a conformal prediction in the binary classification setting, the *confidence* of a prediction is defined as 1 − *p*_2_ where *p*_2_ is the lower *p*-value of the two (Saunders et al., 1999). This means that even if a prediction has one high *p*-value, its confidence and hence usefulness in a decision setting might still be low. Figure 7C shows the target profile for Pergolide, an agonist for DRD1, DRD2, HTR1A, and HTR2A which shows up as the four highest active predictions in the profile.

## 4. Discussion

We have presented a reproducible workflow for building profiles of predictive models for target-binding. We have exemplified our approach on data from ExCAPE-DB about 31 targets associated with adverse effects and made these models available both via a graphical web interface via an OpenAPI interface for programmatic access and made them available for download. The Conformal Prediction methodology guarantees validity of the models under the exchangeability assumption. We have further showed that our models are indeed valid, with the calibration plots in Figure 3.

Based on the efficiency metrics shown in Figures 4B,C we see that the efficiency, after adding assumed non-actives to the datasets with very few (under 10,000) non-actives, is clearly improved. Based on the external test set, Dataset4, though, especially based on the plots in Figure 5, we see that there is a somewhat higher fraction of observed non-actives (“N”) correctly predicted as non-actives, than the fraction of observed actives (“A”) correctly predicted as active.

The use of workflows to automate pre-processing and model training and make it completely reproducible has several implications. Primarily, the entire process can be repeated as data change, e.g., when new data is made available or data is curated. In our case, the pre-processing can be re-run when a new version of ExCAPE-DB is released, and new models trained on up-to-date data can be deployed and published without delay. The components of the pre-processing workflow are however general, and can be re-used in other settings as well. Further, a user can select the specific targets that will be pre-processed, and focus the analysis on smaller subsets without having to pre-process and train models on all targets, which could be resource-demanding. With a modular workflow it is also easy to replace specific components, such as evaluating different strategies and modeling methods.

The packaging of models as JAR-files and Docker containers makes them portable and easy to transfer and deploy on different systems, including servers or laptops on public and private networks without cumbersome dependency management. We chose to deploy our services inside the RedHat OpenShift container orchestration system, which has the benefit of providing a resilient and scalable service, but any readily available infrastructure provider is sufficient. The use of OpenAPI for deploying an interoperable service API means that the service is simple to integrate and consume in many different ways, including being called from a web page, (such as our reference page on http://ptp.service.pharmb.io/) but also into third party applications and workflow systems. With the flexibility to consume models on individual level comes the power to put together custom profiles (panels) of targets. In this work we have selected targets based on usefulness in a drug safety setting, but it is easy to envision other types of panels for other purposes. While there has been some previous research on the use of predicted target profiles (Yao et al., 2016; Awale and Reymond, 2017), further research is needed to maximize their usefulness and to integrate with other types of *in vitro* and *in silico* measures. Our methodology and implementation facilitates such large-scale and integrative studies, and paves the way for target predictions that can be integrated in different stages of the drug discovery process.

## 5. Conclusion

We developed a methodology and implementation of target prediction profiles, with fully automated and reproducible data pre-processing and model training workflows to build them. Models are packaged as portable Java Archive (JAR) files, and as Docker containers that can be deployed on any system. We trained data on 31 targets related to drug safety, from the ExCAPE-DB dataset and published these as a predictive profile, using Conformal Prediction to deliver prediction intervals for each target. The example profile is deployed as an online service with an interoperable API.

## Data availability

- The datasets analyzed for this study can be found on Zenodo (for ExCAPE-DB) (Sun et al., 2017b), and on the DrugBank website (for DrugBank datasets) (Dru, 2018).
- The data (i.e., the predictive models) generated in this study are available on Zenodo at Lampa et al. (2018a).
- Source code used in this study, is available on GitHub at PTP (2018).

## Author contributions

OS conceived the study. OS, JA, SA, and SL designed the study, interpreted results, and wrote the manuscript. SL implemented the workflow and carried out the analysis. SA extended CPSign with new features. JA, SA, and AB contributed with model deployment and APIs. EA contributed with expertise in target profiles and modeling. All authors read and approved the manuscript.

### Conflict of interest statement

OS, JA, AB, and SA are involved in Genetta Soft AB, a Swedish based company developing the CPSign software.

The remaining authors declare that the research was conducted in the absence of any commercial or financial relationships that could be construed as a potential conflict of interest.

## Footnotes

^1^https://github.com/pharmbio/ptp-project/blob/c529cf/exp/20180426-wo-drugbank/wo_drugbank_wf.go#L239-L246

^2^The term “*p-values*” in Conformal Prediction does not have the same definition as in statistical hypothesis testing.

## Abbreviations

A: Active
ACP: Aggregated Conformal Predictor
CAOF: Class-Averaged Observed Fuzziness
CP: Conformal Prediction
JAR: Java Archive (A file format)
MC: M Criterion (Fraction of multi-label predictions)
N: Non-active
OF: Observed Fuzziness
QSAR: Quantitative Structure-Activity Relationship
RF: Random Forest
SMILES: Simplified molecular-input line-entry system (A text-based representation of chemical structures)
SVM: Support Vector Machines.
