# Supplementary material for: Predicting Off-Target Binding Profiles With Confidence Using Conformal Prediction
Source: Front Pharmacol. 2018 Nov 6;9:1256. doi: 10.3389/fphar.2018.01256 (PMC6233526; doi:10.3389/fphar.2018.01256)
Supplement: Supplementary file 1 [file Data_Sheet_1.PDF]

Supplementary Material: Predicting off-target binding profiles with confidence using Conformal Prediction

SUPPLEMENTARY TABLES AND FIGURES

Figures

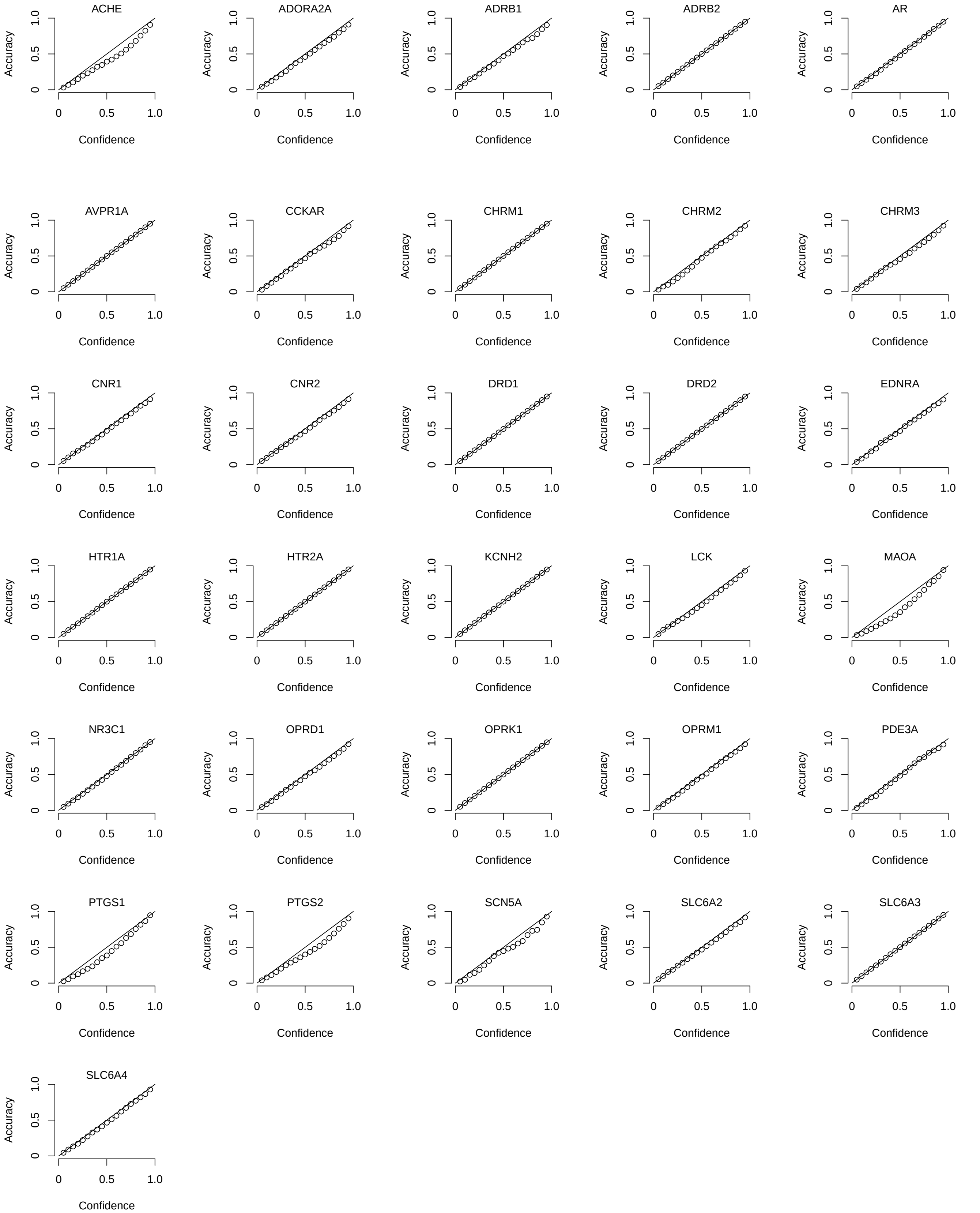

Figure S1: Calibration plots for all targets. The plots show accuracy against confidence, for confidence values 0.05 to 0.95 with a step size of 0.05.

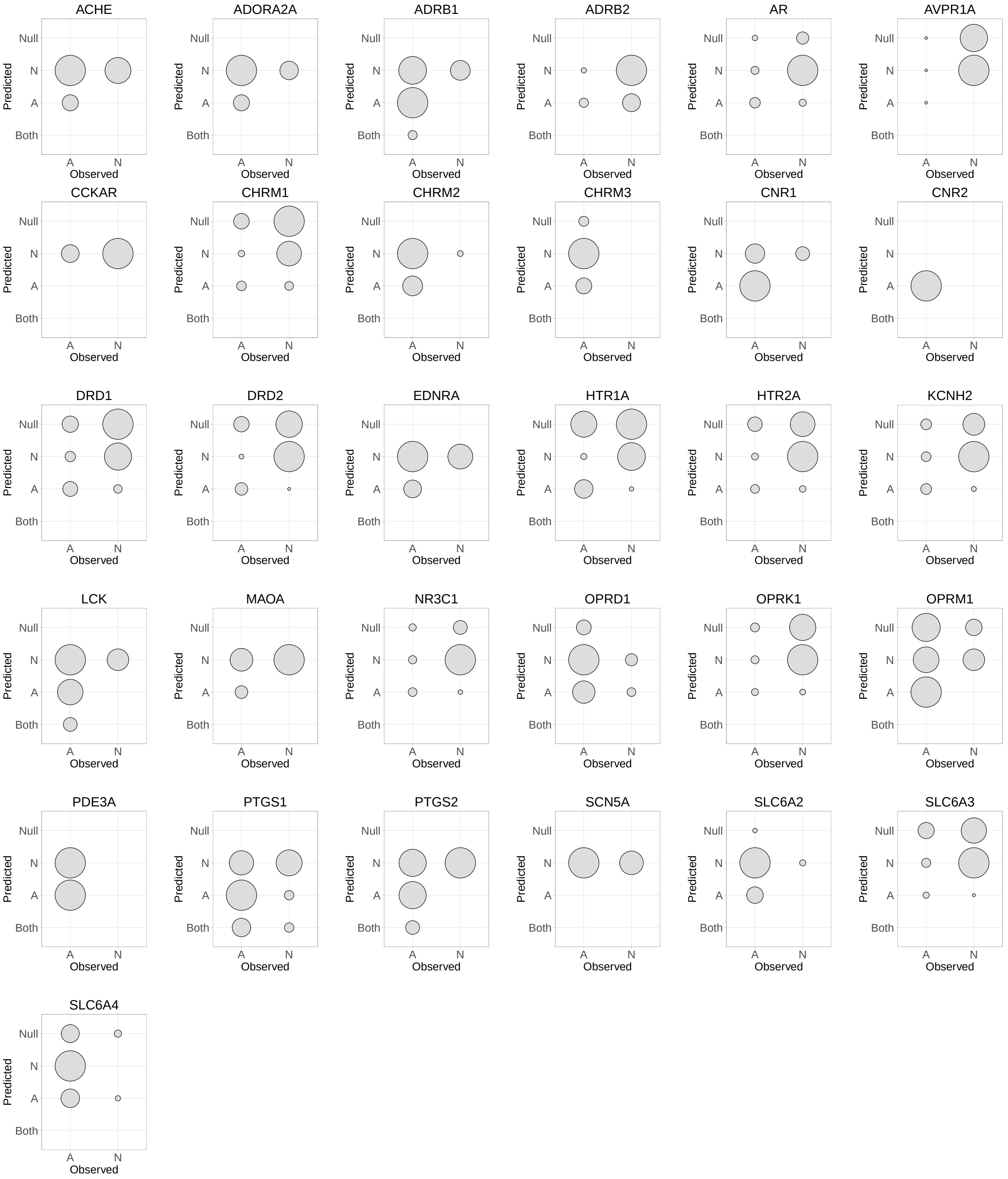

Figure S2: Predicted versus observed labels, at confidence level 0.8, for all targets, and all compounds in the prediction dataset. The X-axis represents observed labels, as found in ExcapeDB, while Y-axis shows predicted labels. The areas of the circles is proportional to the number of compounds per predicted/observed combination. Note that the scale is different between each plot, because of differing total number of compounds per target.

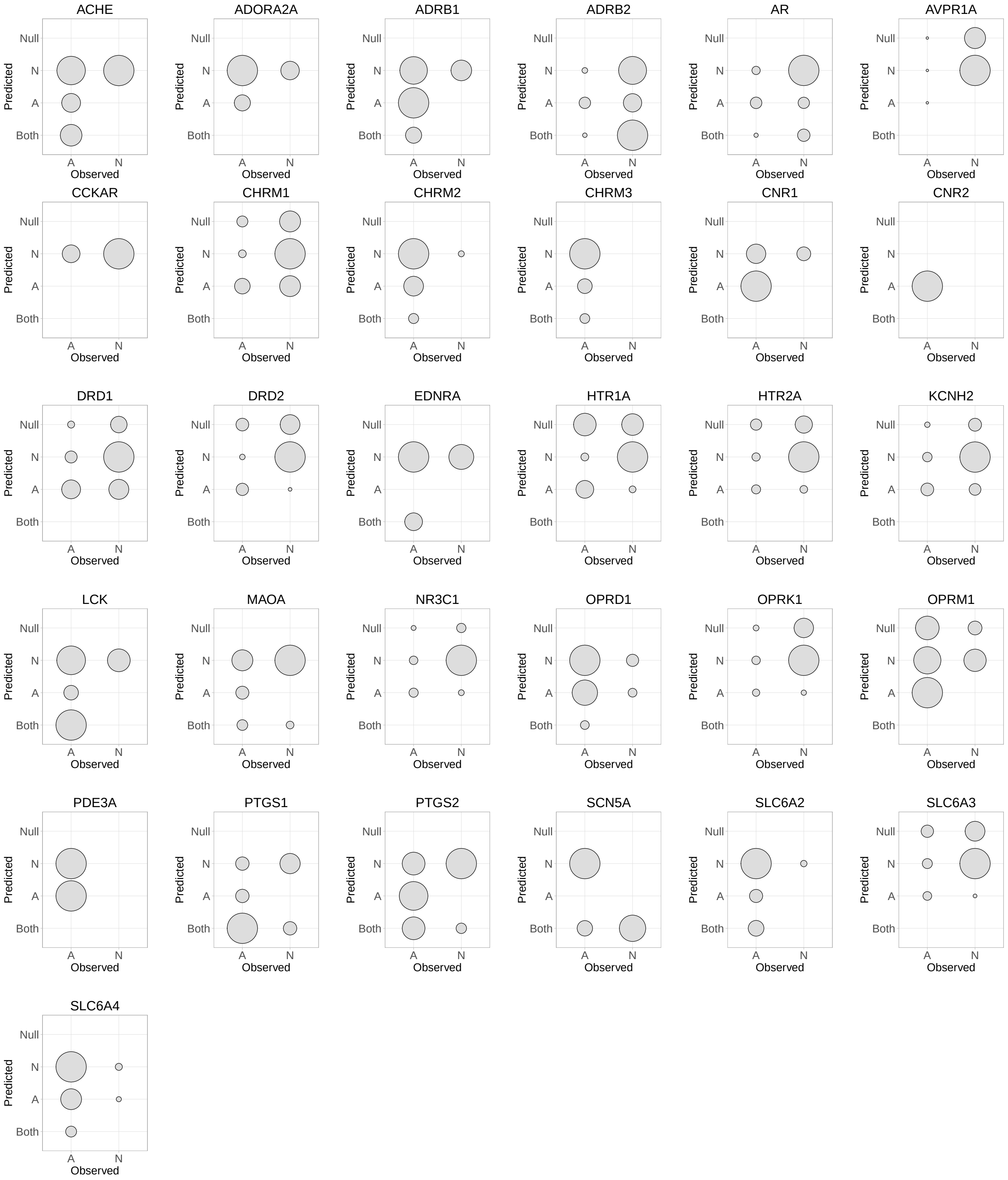

Figure S3: Predicted versus observed labels, at confidence level 0.9, for all targets, and all compounds in the prediction dataset. The X-axis represents observed labels, as found in ExcapeDB, while Y-axis shows predicted labels. The areas of the circles is proportional to the number of compounds per predicted/observed combination. Note that the scale is different between each plot, because of differing total number of compounds per target.

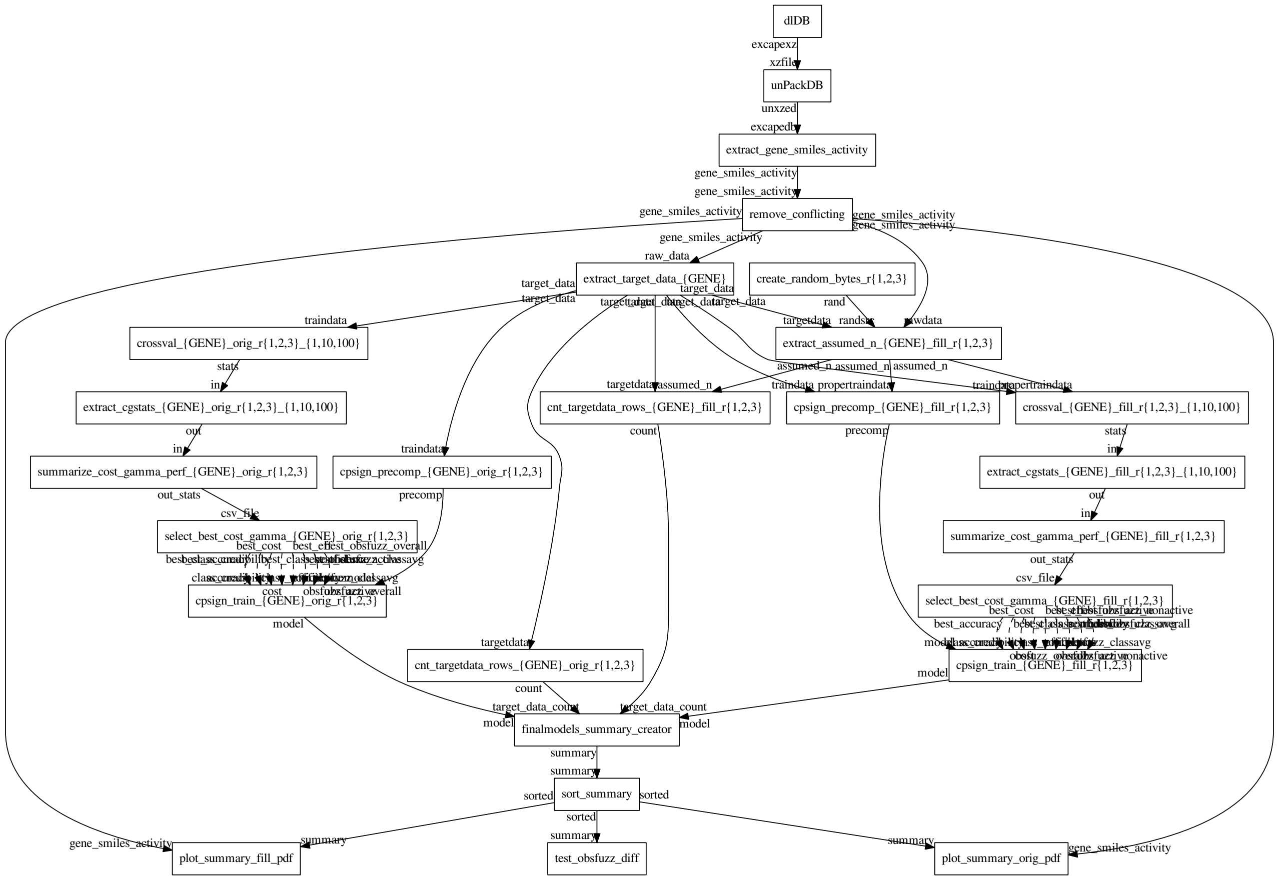

Figure S4: Detailed workflow graph for comparing the effect of extending target datasets with assumed non-actives.

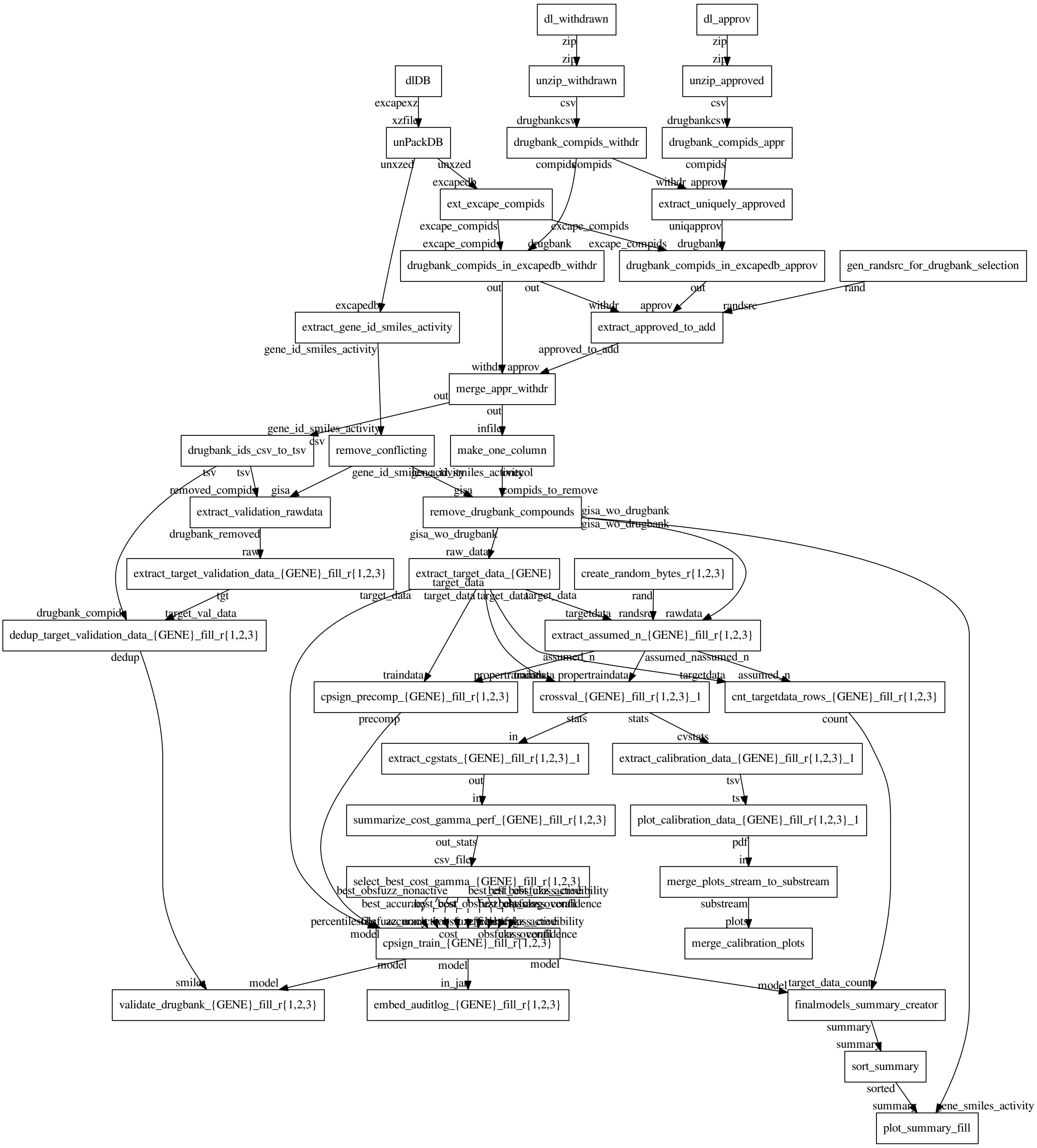

Figure S5: Detailed workflow graph for the workflow where DrugBank compounds were removed. Note the additional components in the top of the figure, for preparing and extracting data from the DrugBank dataset.
